# Polymorphisms of *brain*-*derived neurotrophic factor* genes are associated with anxiety and body mass index in fibromyalgia syndrome patients

**DOI:** 10.1186/s13104-020-05226-8

**Published:** 2020-08-28

**Authors:** Boya Nugraha, Sumadi Lukman Anwar, Christoph Gutenbrunner, Christoph Korallus

**Affiliations:** 1 Department of Rehabilitation Medicine, Hannover Medical School, Hannover, Germany; 2 Department of Surgery, Universitas Gadjah Mada, Yogyakarta, Indonesia

**Keywords:** Fibromyalgia, Single nucleotide polymorphism, Brain-derived neurotrophic factor, Mood symptom

## Abstract

**Objective:**

Fibromyalgia syndrome has been associated with familial clusters although the specific genetic predisposition is not clear. Accordingly, studies concerning genetic factors associated with this disease are important. Brain-derived neurotrophic factor (*BDNF*) has been shown to play a role in patients with fibromyalgia syndrome, particularly in mediating manifestations of pain and mood-related symptoms. Research on genetic factors, including genetic variations or single nucleotide polymorphisms, especially related to *BDNF* in fibromyalgia is very limited. Therefore, this study was aiming at determining the association of polymorphisms of *BDNF*, particularly rs2049046 (A>T) and rs7124442 (A>G), with body mass index (BMI) and mood-related symptoms in FMS.

**Results:**

In fibromyalgia syndrome cases, *BDNF* polymorphisms were associated with body mass index and anxiety score, specifically rs7124442 (A>G) (Fisher’s exact test χ^2^; *p *< 0.05; odds ratio (OR): 1.02) and rs2049046 (A>T) (Fisher’s exact test χ^2^; *p *< 0.05; OR: 0.55), respectively. Additionally, patients with fibromyalgia syndrome who have AA (95% CI (8.71, 11.63)) and AT (95% CI (9.32, 11.74)) alleles of rs2049046 showed higher score of anxiety compared to patients with TT (95% CI (3.98, 8.20) allele (ANOVA test; *p *< 0.01). These results suggest that *BDNF* polymorphisms (rs7124442 and rs2049046) are associated with body mass index and anxiety symptoms in patients with fibromyalgia syndrome.

## Introduction

Etiology and pathogenesis of fibromyalgia syndrome (FMS) are still not clear and need to be elucidated more. In patients with FMS, familial inheritance might have an important role, since FMS is often found in more than one member of a family [1]. Therefore, studies concerning genetic factors associated with this disease are important and relevant. Genetic variations or single nucleotide polymorphisms (SNPs) have been associated with different types of diseases. SNPs also affect the development of diseases [2], response to drug treatment [3] and pathogens [4]. Therefore studies of SNPs on FMS patients could advance the understanding of the pathogenesis of FMS.

Regarding the mode of inheritance in FMS, no specific genes have been reported. Only a few studies have suggested some underlying genetic polymorphisms in FMS patients, such as catechol-*o*-methyltransferase, serotonergic system of 5HTTA [5], and D4 dopamine receptor exon III repeat polymorphism [6]. Another gene of interest in FMS is brain-derived neurotrophic factor (*BDNF*), although the pattern of familial inheritance is not clearly uncovered.

The role of *BDNF* in patients with FMS is already known to be unquestionable, since *BDNF* expression is found to be higher in patients with FMS compared to healthy subjects, both in plasma [7] and serum [8], as well as cerebrospinal fluid [9]. *BDNF* is also known to be related to mental symptoms, such as depression [10] and anxiety [11]. These symptoms are frequently observed in patients with FMS [12]. Additionally, our previous study showed a correlation of *BDNF* and depression in patients with FMS [8].

Therefore, it is important to explore if *BDNF* at the gene level (polymorphism) is also associated with the phenotype manifestations (symptoms) of FMS. This study aimed to examine the association of polymorphisms of *BDNF*, particularly rs2049046 and rs7124442, with several FMS symptoms.

## Main text

### Materials and methods

#### Patients

Seventy-two (n = 72) female FMS patients with age range of 40–70 years were recruited from Rheumatology practices in Hannover, outpatients of the Department of Rehabilitation Medicine of Hannover Medical School and the patient organization “RheumaLiga”, Hannover. Recruitment was also performed by advertising the study in local newspaper. They were screened based on the definition of the American College of Rheumatology (ACR) criteria for fibromyalgia [13] by physicians of the Department of Rehabilitation Medicine, Hannover Medical School, Hannover, Germany. Patients had to understand the German language. Patients were not restricted to have their treatments either pharmacological or non-pharmacological.

Patients with recent or past history of psychiatric disorders, inflammatory, endocrine or other clinically significant chronic diseases (e.g., diabetes, rheumatoid arthritis, inflammatory bowel disease), abnormal liver function, pregnant and breastfeeding women were excluded.

#### Healthy controls (HCs)

HCs should be in the range of 18–70 years old. Forty-two (n = 42) female HCs in the age range of 25–68 years old were recruited and selected. Most of them were staffs of Hannover Medical School, Germany. Exclusion criteria: chronic pain, psychiatric disorders, pregnancy and breastfeeding women.

#### Questionnaires

All patients and HCs completed pain intensity (Visual Analogue Scales; VAS [14]), intensity of fatigue (VAS [8]), and depression and anxiety questionnaires (Hospital Anxiety and Depression Scale; HADS [15]).

#### Patients grouping

Subgroups of patients were assessed for body mass index (BMI) (18.5–24.9: low; 25.0–29.9: normal; > 30.0; obese) [16], and the HADS-anxiety (HADS-A) and HADS-depression (HADS-D) scores using the classification given by Zigmond and Snaith [15] (HADS-A/HADS-D score < 8: no depression/anxiety; HADS-A/HADS-D score 8–10: mild depression/anxiety; HADS-A/HADS-D score > 10: positive depression/anxiety).

#### DNA extraction and generation of the polymerase chain reaction (PCR) product

Overnight fasting peripheral venous blood samples were collected in EDTA tubes (Monovette^®^, Sarstedt, Germany) from all patients and HCs between 08:00 and 10:00 am. Genomic DNA from whole blood containing EDTA was extracted by using DNeasy^®^ Blood & Tissue Kit (Cat No. 69506, Qiagen) and according to the manufacturer’s instructions.

Prior to pyrosequencing analysis, 50 ng DNA from each sample was amplified with PCR using 0.4 μM pair of primers (Sigma-Aldrich, Darmstadt, Germany), 0.2 mM dNTP, 0.02 unit/L Taq polymerase (Invitrogen, Karlsruhe, Germany), 1× PCR buffer (Invitrogen, Karlsruhe, Germany), and purified HPLC water to add the total volume of 25 μL. The PCR process was optimized for each of the primer sets with final concentration of MgCl_2_ (1.5 mM) and the annealing temperature (60 °C). The amplification was programmed using the following conditions: 15 min at 95 °C; 45 cycles of (94 °C for 30 s; 60 °C for 30 s; 72 °C for 30 s); and 72 °C for 5 min. PCR products were checked using DNA ladder (1 Kb Plus DNA Ladder Invitrogen) in 4% acrylamide gel and visualized by using UV light after incubation with ethidium bromide for 30 min for amount and specific sizes of 129 bp for rs2049046 and 140 bp for rs7124442, respectively. Sets of primers were designed using PyromMark Assay Design SW 1.0 (Qiagen, Hilden, Germany).

Primers for the two *BDNF* SNPs:

rs2049046

Forward primer 5′- 3′ TCACCCAGGTGATTTTTATGC

Reverse primer 5′- 3′ CCAATTTGTGCAGACCTTAAA

rs7124442

Forward primer 5′- 3′ TGTCCCTCAAAAGGAAGCTG

Reverse primer 5′- 3′ TGTAGATTTGTTTTGTGTTGTTTGAA

Pyrosequencing

Pyrosequencing analysis was done as described by Anwar et al. [15] using PyroMark Q96 (Qiagen, Hilden, Germany). Strand separation was done using streptavidin Sepharose^®^ (SigmaAldrich, Darmstadt, Germany) using PyroMark Q96 Vacuum Workstation (Qiagen, Hilden, Germany).

The following pyrosequencing primers were used:

rs2049046: pyrosequencing primer 5′- 3′ TGCTCCGAGGAGGTCC

rs7124442: pyrosequencing primer 5′- 3′ ATGCTTGGAATATCTGC

Pyrograms were analyzed using PSQ 96MA SNP/Pyromark ID Software (Qiagen, Hilden, Germany) to determine the genotypes of each sample (Additional file 1: Figure s1). We also performed Sanger sequencing for samples with polymorphisms of rs2049046 (A/T) and rs7124442 (A/G) as a validation.

#### Statistical analysis

T-tests were used to compare demographic and clinical features of HCs and patients with FMS. Chi square test or Fischer’s exact test was used to test (a) genotype and allele distribution, (b) genotype distribution in the subgroups of BMI, anxiety and depression. ANOVA was used to compare any significance of clinical features among genotype distribution. Results with *p *< 0.05 were considered statistically significant using SPSS 19.0 (IBM Corp., Chicago). p values for Hardy–Weinberg equilibrium were also determined.

### Results

Table 1 shows demographic and clinical features of HCs and patients with FMS. FMS patients have significantly higher scores in pain, fatigue, anxiety, depression. The BMI of patients with FMS is also higher as compared to HCs. The tender points of FMS patients in this study were 15. The patients also have had this disease for about 7 years.

**Table 1:** Demographic of HC and FMS patients

Variables	HCs (n = 42)	FMS patients (n = 72)	*p*^#^
Age (years-old)	53.12 ± 0.83	54.19 ± 1.10	0.495
Pain (VAS)	0.95 ± 0.20	6.11 ± 0.21	< 0.000
Fatigue (VAS)	1.80 ± 0.36	6.36 ± 0.23	< 0.000
Anxiety (HADS-A)	4.62 ± 0.56	9.75 ± 0.44	< 0.000
Depression (HADS-D)	2.81 ± 0.50	8.16 ± 0.46	< 0.000
BMI (kg/m^2^)	24.80 ± 0.57	27.70 ± 0.63	0.001
No. tender points	–	15.39 ± 0.27	–
Disease duration (years)	–	7.29 ± 0.59	–

BMI body mass index, *FMS* fibromyalgia syndrome, *HADS* Hospital Anxiety and Depression Scale, *HCs* healthy controls, *VAS* Visual Analogue Scales; Data presented as mean ± SEM

^#^*t* test

The genotype and allele frequency distributions of two different types of *BDNF* SNPs (rs2049046 and rs7124442) are shown in Table 2. The genotype distribution and allele frequency of these *BDNF* SNPs were not significantly different between HCs and patients with FMS (rs2049046: p > 0.05; Odds ratio (OR): 0.55; rs7124442: p > 0.05; OR: 1.02). Additionally, the Hardy–Weinberg equilibrium of rs2049046 and rs7124442 in both patients and HC were not significantly different.

**Table 2:** Genotype and allele frequency distribution of BDNF SNPs in HCs and FMS patients

Group		Genoytpes				Allele	
		rs2049046			p HWE^#^	rs2049046	
		AA	AT	TT		A	T
HC (n = 42)	n	8	27	7	0.063	43	41
FMS (n = 72)	n	26	33	13	0.656	85	59
χ^2^		*p* > 0.05^$^				*p* > 0.05^$^	
		OR: 0.55					

		rs7124442			p HWE^#^	rs7124442	
		AA	GA	GG		A	G
HC (n = 42)	n	21	19	2	0.373	41	23
FMS (n = 72)	n	38	28	6	0.794	104	40
χ^2^		*p* > 0.05^$^				*p* > 0.05^$^	
		OR: 1.02					

*HWE* Hardy–Weinberg equilibrium, *OR* odds ratio

^$^χ^2^ Fisher’s exact test

^#^Chi-square test

Table 3 shows the genotype distribution of each of the *BDNF* SNPs and clinical scores in patients with FMS. Significant difference was found in rs2049046 in anxiety score, but no differences in other clinical symptoms, such as pain fatigue, number of tender points, BMI and depression. FMS patients with lower anxiety score have TT genotype at rs2049046. In rs7124442, there were no significant differences in all clinical symptoms. Table 3 also shows the genotype distribution of the two *BDNF* SNPs, patients with FMS were sub-grouped according to their BMI, anxiety and depression scores. Significant differences were found in BMI of rs7124442 and anxiety of rs2049046, but not in the subgroup of depression.

**Table 3:** Genotype distribution of two *BDNF* SNPs of FMS and its clinical features

Clinical feature scores based on genotype distribution of *BDNF* SNPs								
	rs2049046				rs7124442			
	AA (n = 26)	AT (n = 33)	TT (n = 13)	*p*	GG (n = 6)	GA (n = 28)	AA (n = 38)	*p*
Pain	6.0 ± 0.4	5.4 ± 0.3	5.9 ± 0.6	N.S	6.5 ± 0.6	5.7 ± 0.4	5.6 ± 0.3	N.S
Fatigue	5.8 ± 0.4	6.0 ± 0.4	6.2 ± 0.6	N.S	5.0 ± 1.0	6.0 ± 0.4	6.1 ± 0.3	N.S
No. tender points	14.5 ± 0.6	15.4 ± 0.5	14.6 ± 0.8	N.S	15.5 ± 0.9	15.2 ± 0.5	14.6 ± 0.5	N.S
BMI	27.2 ± 1.1	27.6 ± 0.9	29.1 ± 1.5	N.S	24.7 ± 1.4	29.4 ± 1.1	26.9 ± 0.8	N.S
Anxiety	10.0 ± 0.8	10.4 ± 0.6	7.4 ± 1.1	< 0.002^§^	12.2 ± 1.9	9.6 ± 0.7	9.4 ± 0.6	N.S
Depression	8.3 ± 0.8	8.5 ± 0.7	7.5 ± 1.1	N.S	9.2 ± 1.4	7.8 ± 0.8	8.4 ± 0.6	N.S

Genotype distribution of two *BDNF* SNPs in different subgroups of clinical phenotypes								
rs2049046					rs7124442			
BMI	AA	AT	TT		BMI	AA	GA	GG
18.0–24.9	11	12	3		18.0–24.9	19	5	3
24.9–29.9	9	11	6		24.9–29.9	10	13	3
> 30	6	10	4		> 30	9	10	0
	N.S					*p *= 0.032^#^		

HADS-A	AA	AT	TT		HADS-A	AA	GA	GG
< 8	7	3	6		< 8	9	6	1
8–10	8	14	5		8–10	14	11	2
> 10	11	16	2		> 10	16	10	3
	*p *= 0.048^#^					N.S		

HADS-D	AA	AT	TT		HADS-D	AA	GA	GG
< 8	12	14	8		< 8	20	12	2
8–10	5	9	1		8–10	5	9	1
> 10	10	9	4		> 10	14	5	4
	N.S					N.S		

*SNP* single nucleotide polymorphisms, *BMI* body mass index, *HADS-A* HADS-anxiety, *HADS-D* HADS-depression, *N.S* not significant

^§^ANOVA test

^#^χ^2^ Fisher’s exact test

### Discussion

Pathogenesis of FMS is still not clear and need to be elucidated further. Studying genetic abnormalities in this group of patients are important and relevant. Thus, the purpose of the study was to elucidate the abnormalities of *BDNF* in patients with FMS from a genetic perspective. *BDNF* has been known to play a role in the neuronal system, including neuronal development and synaptic functions. Polymorphisms or variants of *BDNF* are associated with different types of diseases which reflect its involvement in neuronal function. The association of *BDNF* in chronic pain, including patients with FMS has been well-known. In this study, we observed no significant differences of BDNF between patients with FMS and healthy subjects at SNPs rs2049046 and rs7124442, which had not been determined previously in FMS. Some association has already been found in other patients with chronic pain (migraine) involving the SNP rs2049046 [17]. Interestingly, when we focused only on the FMS population, significant differences were observed in the BMI of rs7124442 and anxiety subgroup of rs2049046.

In FMS group, patients with AA homozygous and AT heterozygous at rs2049046 demonstrated higher anxiety scores compared to TT homozygous at rs2049046. Meanwhile, in a study of migraine populations both genotype and allele frequencies of *BDNF* rs2049046 were not significantly different [17], although the heterozygous AT genotype at this SNP was associated with the increased risk of migraine [18]. Regarding the mood/stress related situation, rs2049046 has been seen to show similar moderating effects on social stress-sensitivity [19].

It has been known that high score of BMI is associated with chronic pain, including FMS [19]. Even, obese subjects have higher chances of having FMS by 56% as compared to normal body weight individuals [20]. Additionally, obesity is also affected the severity of symptoms and disease activity in patients with FMS [19]. Through several studies, it has been shown that *BDNF* is associated with patients’ BMI in different types of health conditions [20–23]. Therefore, subgrouping of patients according to their BMI was performed and investigated in this study. This hypothesis was approved in our result that BDNF rs712442 is associated with BMI.

The *BDNF* SNP at rs7124442 is located in the 3′-untranslated region (UTR) of the *BDNF* gene. The polymorphism at this site seems to influence the activity-dependent *BDNF* mRNA targeting, translation and/or degradation at active synaptic sites as well as the proBDNF transcript [25, 26]. *BDNF* rs7124442 SNP has been shown to be functionally related to BDNF plasma levels in subjects with eating disorders [24]. This association has also been examined previously with respect to Alzheimer’s disease [28]. Increased risk for major depression in two- and three-loci gene interactions in a Chinese population for SNPs rs7124442 and rs6265 has been observed [25], although in our FMS population, we could not observe the association of rs7124442 with mood related disorders.

The most studied *BDNF* variant, in general, is rs6265. Reflecting its function, this SNP has been reported in FMS patients with differences reflected in their BMI [27], which may be the only *BDNF* variation study in patients with FMS. Moreover, there are neuroplastic effects of mature BDNF that are also affected [28], which are suggested to be associated with several diseases, including major depression [29], anxiety [30], and hypothalamus–pituitary–adrenal-axis activity [31]. Therefore, our results according to our knowledge for both SNPs at rs7124442 and rs2049046 are new in the FMS population.

## Conclusions

These results suggest that *BDNF* polymorphisms are associated with body mass index (rs7124442) and anxiety (rs2049046) in patients with FMS.

## Limitations

One limitation in this study was the small sample size which may be the cause of our finding no significant differences for both BDNF SNPs between patients with FMS and HCs. More studies with larger numbers of samples are still needed to elucidate the role of genetic variations of BDNF in patients with FMS with further consideration of other factors, such as sex and the age of the patients. However, our results could observe some interesting findings in the FMS patient subgroups such as significant association of BMI and anxiety score with BDNF SNP at rs2049046 and rs7124442, respectively. This report is an exploratory study that could give important insights for further research, e.g. for sample size calculation for larger studies. Therefore, our current findings should be carefully extrapolated to the general population.

## Supplementary information

**Additional file 1: Figure S1.** Pyrograms from individuals with polymorphisms of rs712442 and rs2049046. Representative pyrograms from a HC with AT allele and a FMS patient with AT allele of rs2049046 were shown in panel A and B, respectively. Representative pyrograms from a HC with GA allele and a FMS patient with AA allele of rs712442 were shown in panel C and D, respectively.

## Data Availability

Raw data are available from the corresponding author on reasonable request.

## Abbreviations

SNPs: Single nucleotide polymorphisms
FMS: Fibromyalgia syndrome
BDNF: Brain-derived neurotrophic factor
HC: Healthy control
VAS: Visual Analogue Scales
HADS: Hospital Anxiety and Depression Scale
HADS-A: HADS-anxiety
HADS-D: HADS-depression
PCR: Polymerase chain reaction
BMI: Body mass index
N.S: Not significant
UTR: 3′-untranslated region
