# Supplementary material for: Polymorphisms of brain-derived neurotrophic factor genes are associated with anxiety and body mass index in fibromyalgia syndrome patients
Source: BMC Res Notes. 2020 Aug 28;13:402. doi: 10.1186/s13104-020-05226-8 (PMC7456381; doi:10.1186/s13104-020-05226-8)
Supplement: Supplementary file 1 — Additional file 1: Figure S1. Pyrograms from individuals with polymorphisms of rs712442 and rs2049046. Representative pyrograms from a HC with AT allele and a FMS patient with AT allele of rs2049046 were shown in panel A and B, respectively. Representative pyrograms from a HC with GA allele and a FMS patient with AA allele of rs712442 were shown in panel C and D, respectively. [file 13104_2020_5226_MOESM1_ESM.docx]

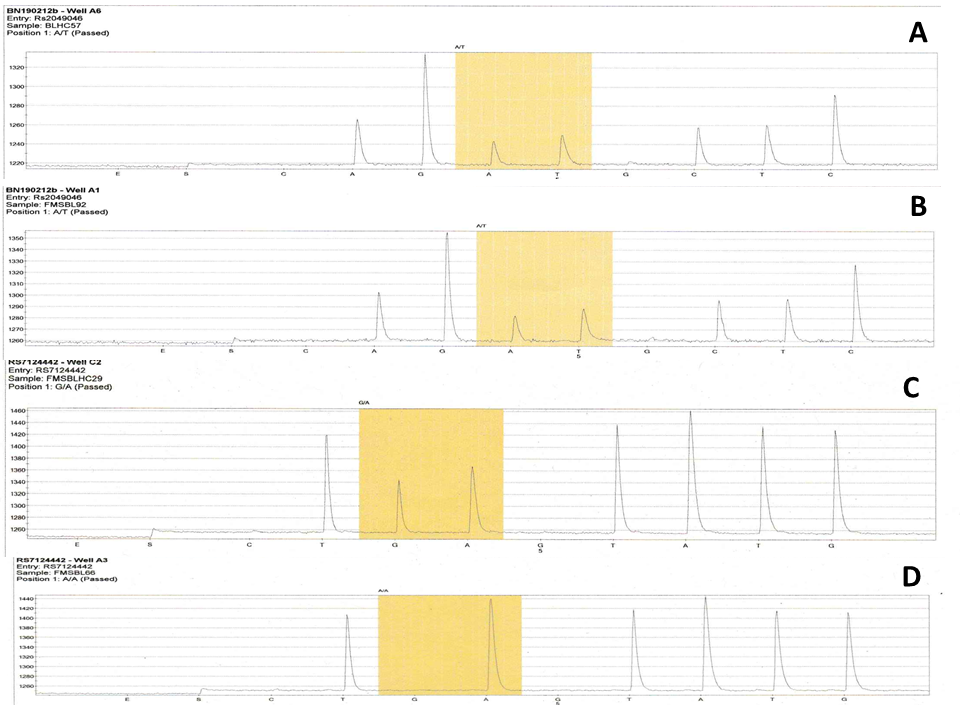


Figure 1. Pyrograms from individuals with polymorphisms of rs712442 and rs2049046. Representative pyrograms from a HC with AT allele and a FMS patient with AT allele of rs2049046 were shown in panel A and B, respectively. Representative pyrograms from a HC with GA allele and a FMS patient with AA allele of rs712442 were shown in panel C and D, respectively.
